# Editorial Note: Biochar enhances wheat crop productivity by mitigating the effects of drought: Insights into physiological and antioxidant defense mechanisms

**DOI:** 10.1371/journal.pone.0343106

**Published:** 2026-02-17

**Authors:** The PLOS One Editors

The *PLOS One* Editors issue this Editorial Note because this article [1] was identified as one of a series of submissions for which we have concerns about aspects of the peer review process. Readers are advised to interpret the article [1] with caution.

The Editors have no evidence of any author involvement in the peer review concerns.
